# Interpersonal physiological synchrony is associated with first person and third person subjective assessments of excitement during cooperative joint tasks

**DOI:** 10.1038/s41598-021-91831-x

**Published:** 2021-06-15

**Authors:** Aiko Murata, Keishi Nomura, Junji Watanabe, Shiro Kumano

**Affiliations:** 1 NTT Communication Science Laboratories, NTT Corporation, Atsugi, Japan; 2 Graduate School of Education, The University of Tokyo, Tokyo, Japan

**Keywords:** Psychology, Human behaviour

## Abstract

Interpersonal physiological synchrony has been shown to play important roles in social activities. While most studies have shed light on the effects of physiological synchrony on recognition of the group state, such as cohesion or togetherness, the effect of physiological synchrony on the recognition of emotional experience has not been adequately researched. In this study, we examined how physiological synchrony is associated with first- and third-person emotion recognition during a joint task. Two participants played a cooperative block-stacking game (Jenga), alternating their roles as player and adviser, while their heart rates were recorded. The participants evaluated their own emotional experience for each turn. Bystanders watched the game to evaluate the players’ emotions. Results showed that the players’ subjective excitement increased not only with their own heart rate, but also with increased heart rate synchrony with their adviser. Heart rate synchrony between player and adviser also related to increased intensity in perceived excitement from the bystanders. Given that both first- and third-person emotion recognition can have cumulative impacts on a group, the relationship between physiological synchrony and emotion recognition observed in the present study will help deepen understanding of the psychophysiological mechanisms underlying larger group phenomena such as crowd excitement.

## Introduction

Physiological activities of the autonomic nervous system (ANS) and neural activities, all of which comprise involuntary and automatic responses, tend to synchronise among people when they are sharing experiences such as rituals^1^, joint actions^2–4^, and prosocial games^5^. Several studies have suggested that physiological synchrony results in psychosocial outcomes. For instance, strong heart rate synchrony relates to togetherness^6^, group cohesion^4,7^, and trust in the opponent^3^, and synchrony in electrodermal activity relates to satisfaction with the group^8^.

### Emotional synchrony and subjective emotion

While these studies shed light on the effects of physiological synchrony on the impressions of the group and group members, the effects on individuals’ subjective experiences of emotion have received less attention. In their research on collective emotion, Páez and colleagues recorded the association between subjective emotions of people and their *self-reported* perceived emotional synchrony during a collective gathering. They showed that participation in a folkloric march strengthened positive emotion as well as social integration, and the intensity of the perceived emotional synchrony with others predicted these effects^9^. This indicates that higher perceived emotional synchrony is associated with stronger emotional reactions that are dominant in a given context. The authors posited that their findings support Durkheim’s theory of collective processes, known as *collective emotional effervescence*^9^. In the Durkheim’s model, people attending collective gathering are expected to be interactively influenced by each other’s emotion, and this mutual emotional sharing strengthens their own emotions, resulting in collective emotional effervescence^10^. While the findings by Páez and colleagues are consistent with Durkheim’s model, it is noteworthy that Durkheim also pointed out that such social impacts occur in daily face-to-face interactions, not just in rituals, and are not always consciously perceived^10^.

Given that physiological responses that reflect ANS activities, such as heart rate, skin conductance, and respiration, are closely associated with emotions^11^, spontaneous and automatic synchronisation of ANS activity between interacting people may be a candidate for the unconscious process underlying mutual emotional sharing that strengthens people’s own emotions when they jointly experience emotional events. However, it remains unclear whether the relation between emotional synchrony and increased subjective emotion holds when emotional synchrony is measured physiologically during naturalistic face-to-face interaction. Thus, we recorded heart rate using electrocardiograms (ECGs) to measure the synchrony of ANS activity while two participants performed a joint task, to verify whether the relationship between emotional synchrony and subjective emotional experiences is observed not only at a cognitive level (i.e., self-report), but also at the unconscious physiological level.

### Emotional coherence and emotion recognition of bystanders

Given that humans essentially live in groups, it is important to consider more complex social contexts such as multi-party interactions. Because observations within a two-person interaction do not adequately capture the emotion recognition involved in multi-party interactions, psychologists and neuroscientists have recently shown increased interest in the perceptions and impressions of uninvolved bystanders watching others interact^12,13^. Their research question is, how are the impressions of third-party bystanders formed when they observe people interacting with each other? In this respect, several studies have shown that the emotions of the person who is interacting with another are not only evaluated on the basis of their own emotional expression but are also modulated by the emotional expressions of surrounding others^14–16^. Specifically, Hess and colleagues demonstrated that participants perceived anger and happiness more intensely when target and group members showed the same, compared with incongruent, emotional expression^16^. This indicates that, from the third-person perspective, the emotions of one individual are not perceived independently of the emotions of others interacting with them. From the findings of previous studies, we conjectured that if the interacting people exhibit consistent physiological emotional responses, the emotional intensity perceived by the bystander would be higher. Therefore, we conducted exploratory analyses on how the physiological states of two interacting individuals and their physiological synchrony affect the recognition of their emotions by bystanders.

### Emotion recognition of an interacting partner

In addition to bystanders’ emotion recognition, we also explored that of a person actively interacting with the target. Researchers have suggested that social cognition is fundamentally different when we are actively engaged with the other in a joint project versus when we are mere observers^17^. In line with this argument, there is evidence showing that neural activities during perception of facial expressions are modulated by the direction in which the target person is facing (toward vs. away from the observer)^18^. This finding indicates that neurobiological mechanisms that underlie social cognition may differ depending on whether we are facing and engaging with the other person or merely observing them from the side as bystanders. However, to our knowledge, few studies have addressed the differences in emotion recognition between interacting partners and bystanders in real-time, face-to-face interaction. Considering the possibility that the process of emotion recognition for a person who is actively interacting (i.e., interacting partner) differs from that of third-party bystanders, we examined how physiological factors such as physiological responses and synchrony are associated with emotion recognition of interacting partners.

### Present study

In the present study, we hypothesised that greater physiological synchrony is associated with (1) stronger subjective emotional experience and (2) greater intensity of recognised emotions from bystanders when people jointly experience emotional events in naturalistic face-to-face interaction. Additionally, we explored how physiological responses and synchrony relates to (3) the intensity of recognised emotions from the interacting partner. In our experiment, we recorded participants’ heart rates while they played cooperative Jenga (Tomy Co., Ltd.) to measure physiological synchrony and responses during a joint task. Jenga is a popular block-stacking game in which players each remove one block in turns from a tower constructed of 54 blocks. After removing the block, the player places it on top of the tower, resulting in an increasingly unstable structure. It has been adopted in various studies to evaluate social-interactive behaviour, such as friendship^19^ and the interactive bodily motions of players^20^ during cooperative play. Another study showed that the neurophysiological activities of two people were synchronised when they played cooperative Jenga^2^. Additionally, Jenga can effectively induce ANS activity, as the structure gradually becomes unstable with each removal of a block from the tower. In cooperative Jenga, two participants are required to take turns removing one block and are allowed to talk to one another about strategy and to agree on which block to remove before touching the block. In our study, they were asked to work together to keep the tower from falling during limited turns (i.e., the number of one block removal; 14 turns in total were taken in our study). Thus, they are required to continuously communicate face-to-face to jointly decide which block to remove, alternating their roles as player and adviser for each turn. Players must remove one block at a time from a stacked tower of blocks, taking care not to collapse the entire tower; advisors provide advice to players.

Participants in our study were also instructed to report their subjective emotional experience after each turn in the role of player, and, when in the role of adviser, to estimate their partner’s emotional experience. Since positive emotions (excitement and fun) are considered dominant in cooperative Jenga, we asked participants to report on these emotions. Concurrently, two bystanders watched the player/adviser interactions and were required to estimate the player’s subjective experience. The ratings were made discretely using tablets.

It should be noted that heart rate waveforms comprise two primary rhythmic oscillations, which are based on different psychophysiological mechanisms. One oscillation reflects vagal activity and is related to respiratory frequency, termed respiratory sinus arrhythmia (RSA; > 0.15 Hz). The other occurs in an approximately 10 s cycle, which reflects an oscillation of the sympathetic vasomotor tone and occurs in synchrony with arterial pressure Mayer waves (< 0.15 Hz) (see^21^ for a review). To our knowledge, however, few studies have addressed heart rate synchrony separately for these two frequency bands. As an exception, Wilson and colleagues reported that synchrony in RSA predicted greater negative affect reactivity during marital conflict^22^. However, they focused only on RSA synchrony; given the difference in the relationship between each frequency band and various emotions^11^, it is likely that the findings were still inadequate for addressing the possible differences in the relationship between the heart rate synchrony of each frequency band and emotions. Accordingly, the analyses of heart rate synchrony in the present study were performed separately for these two frequency bands.

## Results

### Analysis of physiological responses

**Figure 1:**
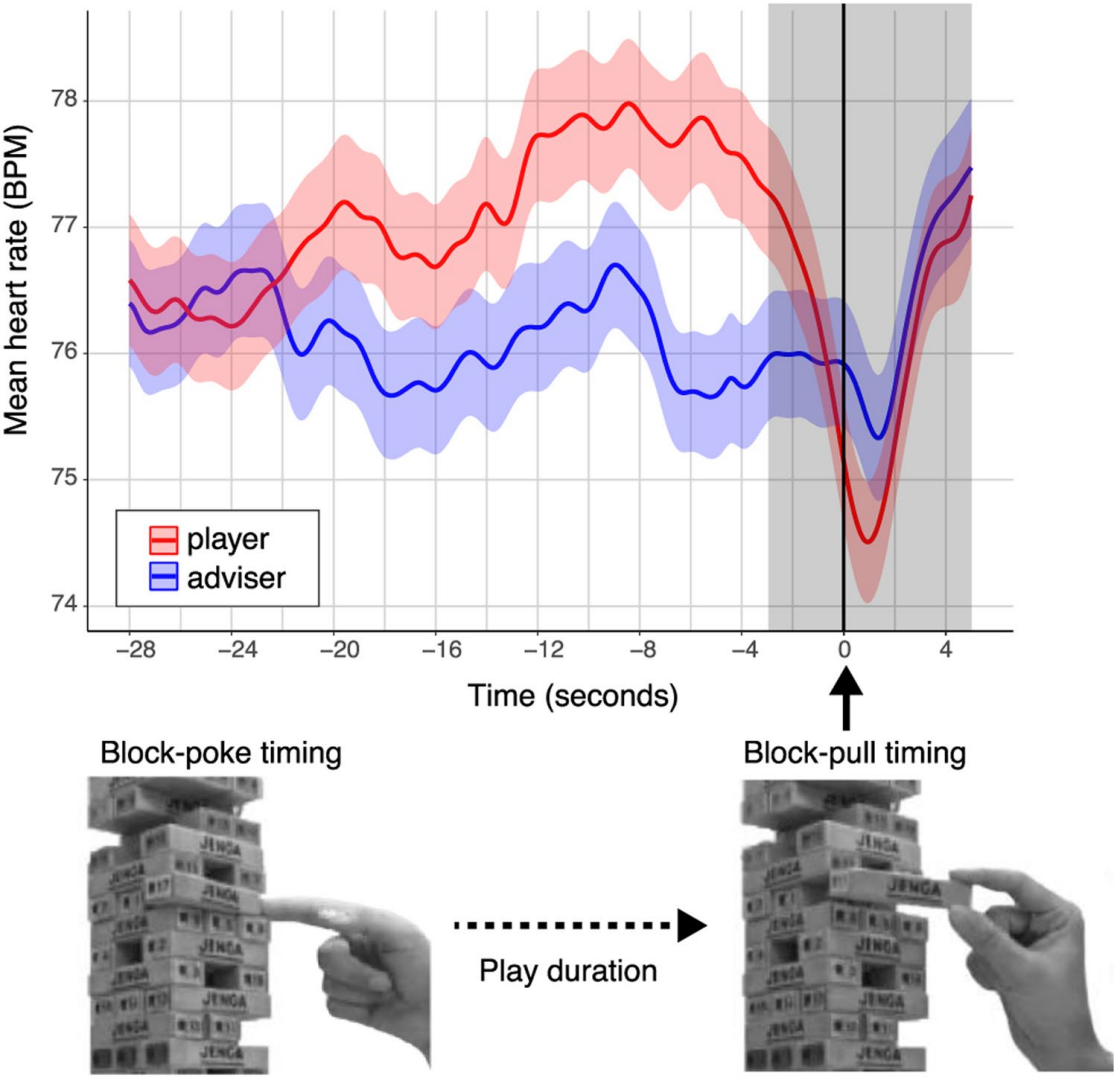
Mean heart rates of players and advisers while playing Jenga. The plot line represents the time series for players’ (*red line*) and advisers’ (*blue line*) mean heart rates (in BPM) while playing Jenga. The x-axis represents the timeline, with time zero seconds marking the start of block-pull timing. The grey shaded area demarcates the time during which heart rates temporarily decreased and then increased (i.e., from 3 s before to 5 s after block-pull timing). It is possible that players and advisers tended to hold their breath for a moment just before the block-pull timing began, which may explain the rapid change in heart rates; these data were not used for subsequent analysis. The duration of play was calculated as an indicator of the difficulty of removing a block for each turn. This was the log-transformed time between when a player successfully removed a block (block-pull timing) and when they first touched that block (block-poke timing, see “Methods” for details).

Figure 1 shows the mean heart rates (in beats per minute, or BPM) for both players and advisers while playing Jenga. Time zero seconds in the figure indicates the beginning of the block-pull timing, which was manually annotated from the recorded video. Specifically, heart rates and inter-beat intervals (IBIs) from 28 s before removing a block to 3 s before removing a block were used for subsequent analysis (i.e., a 25-s period of interest; areas shown in white in Fig. 1).

### Physiological responses of players and advisers during a joint task

As shown in Fig. 1, compared with advisers, the heart rates of players who were required to remove blocks tended to be higher while playing Jenga. To assess the effect of role (player/adviser) on physiological responses, we conducted a linear mixed-effects model (LMM) for mean heart rates during a 25-s period of interest by using the “lmer” function in the “lme4” package for R^23^. In this model, role (player vs. adviser), turn (14turns in total), and their interactions were entered as fixed effects. Considering individual differences in ANS activities for each pair, the pair x player interaction was entered as a random effect. A Wald Chi-Square test using the LMM revealed significant main effects of role (*χ*^*2*^(1) = 4.203, *p* = 0.040) and turn (*χ*^*2*^(1) = 7.823, *p* = 0.005). The interaction was non-significant (*χ*^*2*^(1) = 0.001, *p* = 0.976). The results supported the observations in Fig. 1. Participants’ heart rates were higher when they were players than when they were advisers (*β* = 0.995, *t* = 2.050, *95% CI* = 0.042–1.947), and their heart rates increased with each subsequent turn (*β* = 0.127, *t* = 2.797, *95% CI* = 0.038–0.216).

### Physiological synchrony between player and adviser

Next, we tested whether the physiological responses of player and adviser synchronised while playing Jenga. Interpersonal physiological coherence (IPC) was estimated using wavelet transform coherence (WTC)^24^ as the index of physiological synchrony. WTC is conceptualised as a localised correlation coefficient between two time series as a function of time and frequency^25,26^. The resulting WTC values varied between 1 and 0, representing a perfect synchronisation and no synchronisation, respectively.

**Figure 2:**
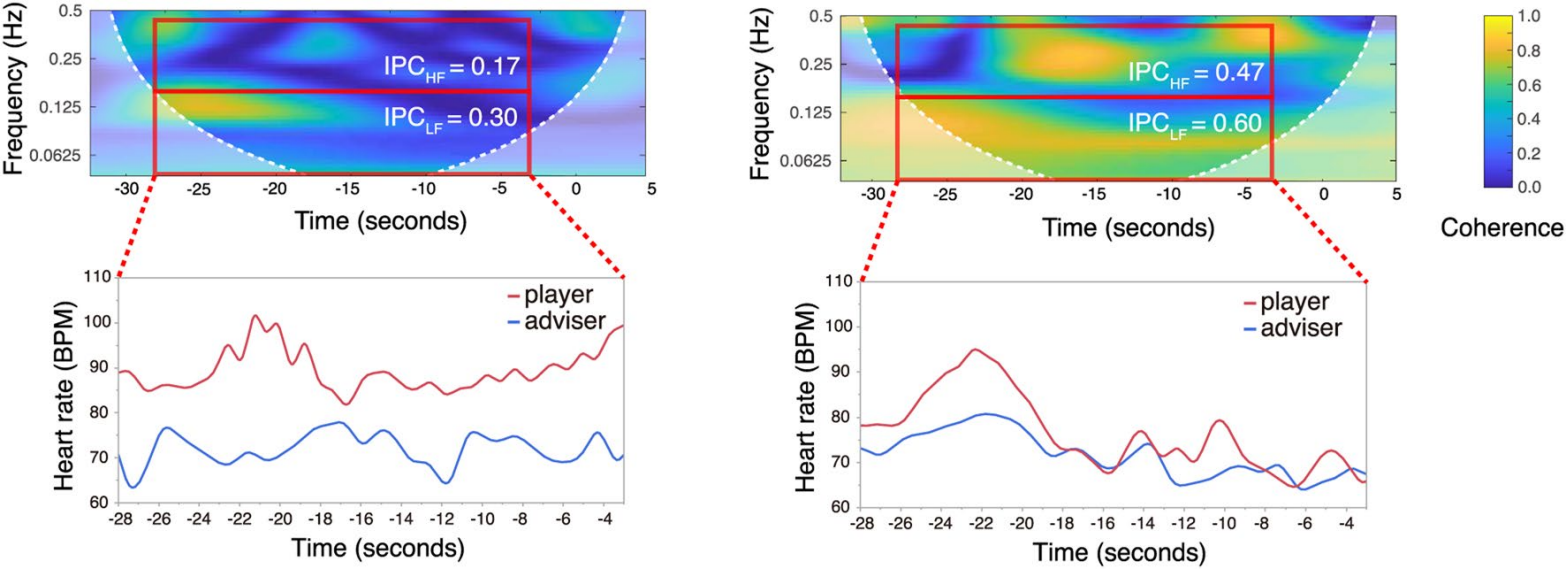
Examples of wavelet transform coherences (WTCs) and interpersonal physiological coherence (IPC). Sample WTCs between a player’s and an adviser’s IBIs in the top images, and a time-series of heart rates for a player (*red lines*) and an adviser (*blue lines*) in the bottom images. All x-axes represent the timeline, with time zero seconds marking the start of block-pull timing (see Fig. 1 for details). The wavelet coherence is represented by colour. The plot on the left is an example of a turn with relatively weak synchrony, and the plot on the right shows a turn with relatively strong synchrony. The *squares outlined in red* in the WTC plots show coherence values for the 25-s period of interest in each of the high (upper squares: 0.15–0.40 Hz) and low-frequency bands (lower squares: 0.04–0.15 Hz). IPC was calculated by averaged coherence values in each square (top squares: IPC_HF_; bottom squares: IPC_LF_); however, the cone of influence (COI) areas of the WTCs, which are a lighter shade, were not included in the IPC calculations.

For the purpose of evaluating whether the heart rates of player and adviser synchronised in a high-frequency band and/or low-frequency band, the WTC of the two IBIs time-series (i.e., player’s IBIs and adviser’s IBIs) was divided (high-frequency band: 0.15–0.40 Hz, low-frequency band: 0.04–0.15 Hz; see Fig. 2). To determine whether IPC was stronger owing to social interaction between two paired participants (i.e., face-to-face interaction in the same session), we compared the IPC measurements of actual pairs with those of pseudo pairs (i.e., two participants from different sessions, see Supplementary Fig. S1 for details).

**Figure 3:**
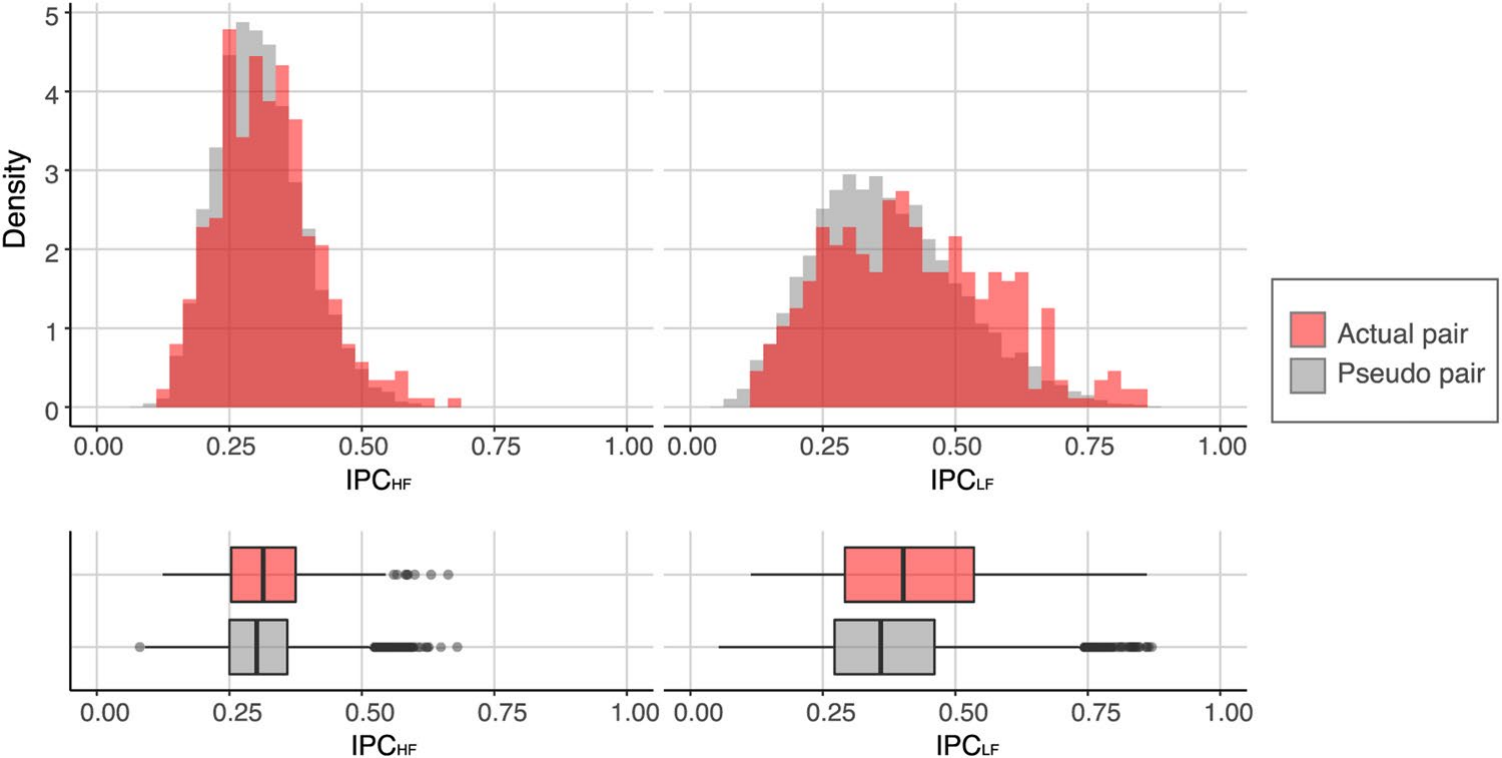
Density plots and box plots for interpersonal physiological coherences (IPCs) in the high-frequency band (IPC_HF_) and low-frequency band (IPC_LF_) as a function of pair type. The red bars represent IPCs of actual pairs. The *grey bars* represent the IPCs of pseudo pairs. The pseudo pairs included all of the possible combinations of two participants who took part in different sessions.

Figure 3 shows the density plots and box plots for the IPC_HF_ and IPC_LF_ as a function of pair type (actual pair vs. pseudo pair). To examine whether the IPC of the actual pairs was stronger than that of the pseudo pairs in both high and low-frequency bands, an LMM analysis for IPC was conducted. In the model, pair type (actual pair vs. pseudo pair), frequency band (high vs. low), turn, and the pair type x frequency band interaction were entered as fixed effects. Considering pair differences in IPC, pair was entered as a random effect. A Wald Chi-Square test using the LMM revealed significant main effects of pair type (*χ*^*2*^(1) = 3.856, *p* = 0.050), frequency band (*χ*^*2*^(1) = 1354.220, *p* < 0.001), and pair type x frequency band interaction (*χ*^*2*^(1) = 15.386, *p* < 0.001). The main effect of turn was not significant (*χ*^*2*^(1) = 0.388, *p* = 0.534). The model showed that IPCs in the low-frequency band (IPC_LF_) were stronger than those in the high-frequency band (IPC_HF_) (IPC_LF_ minus IPC_HF_: *β* = 0.064, *t* = 36.800, *95% CI* = 0.061–0.068), and the IPCs of actual pairs were stronger than those of the pseudo pairs (IPC_actual_ minus IPC_pseudo_: *β* = 0.013, *t* = 1.964, *95% CI* = 0.000–0.026). Additionally, pair type x frequency band interaction in the model indicated that the effect of pair type was different in the high- versus low-frequency bands.

To see the interaction effects in detail, we conducted separate post-hoc LMM analyses for each frequency band, in which turn and pair type were entered as fixed effects and pair was entered as a random effect. The models showed that both IPC_HF_ and IPC_LF_ were different between actual and pseudo pairs, while the effect of pair type on IPC_LF_ (*β* = 0.047, *t* = 6.252, *95% CI* = 0.032–0.062) was relatively large compared with its effect on IPC_HF_ (*β* = 0.013, *t* = 2.713, *95% CI* = 0.004–0.022). Because the IPCs of actual pairs were stronger compared with those of pseudo pairs, we assumed that the physiological synchrony of heart rates between players and advisers increased because of real-time, face-to-face interaction and/or sharing the same activities. Moreover, this effect was evident in the low-frequency band compared with the high-frequency band.

Analysis of IPCs confirmed that heart rates of players and advisers synchronised while playing cooperative Jenga in both low- and high-frequency oscillations; we therefore examined the relationship between emotion recognition and heart rate synchrony in both frequency oscillations.

### Relationship between physiological synchrony and subjective excitement of the first person

To test whether first-person subjective emotional experience is associated with physiological synchrony of two interacting people, we estimated the effects of IPC_HF_ and IPC_LF_ on players’ subjective excitement using LMM analysis. To evaluate the effect of IPC on subjective excitement, it was necessary to control other factors that may influence intensity of excitement, such as physiological arousal and variability. Furthermore, excitement can be influenced not only by physiological states but also by game situations, such as play duration, turn, and the session (a total of six sessions were held per day, with four women participating; see Fig. S1 for details). Therefore, we entered them, as well as IPCs, as fixed effects in the LMM model for a player’s subjective excitement.

The model included indices for physiological synchrony (i.e., IPC_HF_ and IPC_LF_), mean and standard deviation for heart rate of the player and the adviser, play duration, and turn as fixed effects. Given the individual differences in subjective excitement for each pair, the pair x player interaction was entered as a random effect. A Wald Chi-Square test using the LMM revealed the significant main effects of turn (*χ*^*2*^(1) = 57.212, *p* < 0.001), play duration (*χ*^*2*^(1) = 34.522, *p* < 0.001), their own heart rate (player’s mean heart rate: *χ*^*2*^(1) = 6.177, *p* = 0.013), and physiological synchrony in the low-frequency band (IPC_LF_: *χ*^*2*^(1) = 4.303, *p* = 0.038), with no other main effects reaching the threshold for statistical significance (all *p* > 0.05).

The model showed that players’ subjective excitement increased across turns (*β* = 0.191, *t* = 7.564, *95% CI* = 0.141–0.241) and with longer play duration (*β* = 0.604, *t* = 5.876, *95% CI* = 0.402–0.806). As an effect of physiological activity, a player’s subjective excitement increased with their heart rate (*β* = 0.057, *t* = 2.485, *95% CI* = 0.012–0.103), and with higher physiological synchrony between player and adviser in the low-frequency band (*β* = 1.428, *t* = 2.074, *95% CI* = 0.070–2.783).

The results showed that players’ subjective excitement was associated not only with their own physiological arousal and game situations, but also with physiological synchrony between the pair. It should also be noted that the relationship between subjective excitement and physiological synchrony was observed in low- (IPC_LF_), but not in high-frequency bands (IPC_HF_).

### Relationship between physiological synchrony and bystanders’ third-person recognition of excitement

Next, to understand whether bystanders’ emotion recognition is also associated with the physiological synchrony of two interacting people, we conducted an LMM analysis to assess bystanders’ estimation of the players’ excitement. Players’ subjective excitement was added as a fixed effect to identify factors that uniquely affected the bystanders’ emotion recognition. The other fixed effects were identical with those in the model for first-person subjective excitement.

In the model for bystanders’ excitement recognition, in addition to IPC_HF_ and IPC_LF_, means and standard deviations of players’ and advisers’ heart rates, play duration, turn, session, and players’ subjective excitement, were entered as fixed effects. Given the individual differences in perceived excitement for each pair, the pair x player interaction and bystander were entered as random effects. A Wald Chi-Square test using the LMM revealed the significant main effects of the player’s subjective excitement (*χ*^2^(1) = 49.132, *p* < 0.001), turn (*χ*^2^(1) = 56.682, *p* < 0.001), play duration (*χ*^2^(1) = 15.982, *p* < 0.001), the player’s mean heart rate (*χ*^2^(1) = 8.480, *p* = 0.004), and physiological synchrony in the low-frequency band (IPC_LF_: *χ*^2^(1) = 6.653, *p* = 0.010). No other main effects reached the threshold for statistical significance (all *p* > 0.05).

The model showed that a bystander’s estimation of a player’s excitement was positively related to a player’s subjective excitement (*β* = 0.242, *t* = 7.009, *95% CI* = 0.173–0.310). This indicates that the player’s subjective excitement and the bystander’s estimation of it was generally congruent. As in the model for players’ subjective excitement, the intensity of bystanders’ estimation of players’ excitement increased across turns (*β* = 0.140, *t* = 7.529, *95% CI* = 0.103–0.176), with players’ higher heart rates (*β* = 0.039, *t* = 2.912, *95% CI* = 0.013–0.066), and longer play duration (*β* = 0.290, *t* = 3.998, *95% CI *= 0.147–0.434). Furthermore, the greater the physiological synchrony between player and adviser in the low-frequency band, the stronger the estimated excitement was from bystanders (*β* = 1.211, *t* = 2.579, *95% CI* = 0.290–2.133). Given the observed effect of physiological synchrony on bystanders’ excitement recognition, despite players’ subjective excitement score being controlled for, physiological synchrony was clearly associated with perceived excitement from a third-person perspective.

As shown in the model for players’ subjective experiences, bystanders’ estimations of players’ excitement increased with their own heart rate and across turns. Moreover, physiological synchrony in low-frequency bands was associated with bystanders’ estimation of players’ excitement, as was the case with players’ subjective excitement, while the effect of physiological synchrony in high-frequency bands was not observed.

### Relationship between physiological synchrony and advisers’ second-person recognition of excitement

Considering the possibility that the process of emotion recognition for interacting partner differs from that of bystanders, we conducted exploratory analysis of the physiological factors associated with an advisor’s estimation of a player’s emotion experience.

In the model for estimating advisers’ perceptions of players’ excitement, the fixed effects were identical to those in the model for bystanders’ estimations; the pair x adviser interaction was entered as a random effect. A Wald Chi-Square test using the LMM revealed significant main effects of the player’s subjective excitement (*χ*^2^(1) = 8.933, *p* = 0.003), turn (*χ*^2^(1) = 44.282, *p* < 0.001), play duration (*χ*^2^(1) = 13.522, *p* < 0.001), and the adviser’s mean heart rate (*χ*^2^(1) = 5.005, *p* = 0.025). No other main effects reached the threshold for statistical significance (all *p* > 0.05).

The model showed that an adviser’s estimation of a player’s excitement was positively related to that player’s subjective excitement (*β* = 0.143, *t* = 2.989, *95% CI* = 0.049–0.237), indicating that the player’s subjective excitement and the adviser’s estimation of it were generally congruent. Consistent with the model for bystanders’ estimation, the intensity of advisers’ estimation of excitement increased across turns (*β* = 0.160, *t* = 6.654, *95% CI* = 0.113–0.208), and with play duration (*β* = 0.351, *t* = 3.677, *95% CI* = 0.162–0.538). However, inconsistent with the model for bystanders’ estimation, the estimated excitement from advisers was higher when the adviser’s heart rate was high (*β* = 0.050, *t* = 2.237, *95% CI* = 0.006–0.094), while neither the effect of the player’s heart rate nor the effect of physiological synchrony in the low-frequency band was statistically different from zero (Player’s heart rate: *β* = 0.040, *t* = 1.853, *95% CI* = − 0.003–0.083, IPC_LF_: *β* = 0.601, *t* = 0.981, *95% CI* = − 0.603–1.805).

The analyses of the relationships between physiological synchrony and excitement recognition from players, bystanders, and advisers revealed that the intensity of the player’s subjective excitement and the excitement perceived by bystanders was higher when the player’s and the adviser’s physiological responses were synchronised. However, the adviser’s estimation of the player’s excitement was independent of physiological synchrony but was positively related to the adviser’s own heart rate.

### Relationship between physiological synchrony and a player’s subjective fun and bystanders’ and advisers’ recognition of a player’s fun

#### Players’ subjective fun

In the model for subjective fun, the fixed effects and random effect were identical to those in the model for subjective excitement. A Wald Chi-Square test using the LMM revealed the significant main effects of turn (*χ*^2^(1) = 51.678, *p* < 0.001) and their own heart rate (*χ*^2^(1) = 9.414, *p* = 0.002), with no other main effects reaching the threshold for statistical significance (all *p* > 0.05). The model showed that players’ subjective fun increased across turns (*β* = 0.122, *t* = 7.189, *95% CI* = 0.089–0.155), as their heart rate increased (*β* = 0.052, *t* = 3.068, *95% CI* = 0.019–0.085). However, physiological synchrony in the low-frequency band and play duration, which were associated with subjective excitement, were not significantly related to subjective fun (IPC_LF_: *β* = 0.326, *t* = 0.702, *95% CI* = − 0.587–1.237, Play duration: *β* = 0.022, *t* = 0.321, *95% CI* = −0.114–0.159).

#### Bystanders’ recognition of fun

In the model for bystanders’ recognition of fun, the fixed effects and random effect were identical to those in the model for bystanders’ excitement recognition. A Wald Chi-Square test using the LMM revealed the significant main effects of turn (*χ*^2^(1) = 42.763, *p* < 0.001), play duration (*χ*^2^(1) = 6.452, *p* = 0.011), and player’s mean heart rate (*χ*^2^(1) = 4.647, *p* = 0.031), with no other main effects reaching the threshold for statistical significance (all *p* > 0.05). The model showed that the intensity of the fun recognised by the bystander increased across turns (*β* = 0.091, *t* = 6.539, *95% CI* = 0.064–0.118) and with players’ heart rate (*β* = 0.023, *t* = 2.156, *95% CI* = 0.002–0.043), consistent with the model for players’ subjective fun. However, a bystander’s estimation of a player’s fun was not significantly related to a player’s subjective fun (*β* = 0.050, *t* = 1.382, *95% CI* = − 0.021–0.122), indicating that the bystander’s estimation of fun was generally incongruent with the player’s subjective fun. Unlike players’ subjective fun, the intensity of the fun recognised by bystanders increased with longer play duration (*β* = 0.134, *t* = 2.540, *95% CI* = 0.031–0.238). Physiological synchrony in the low-frequency band, which was associated with bystanders’ excitement recognition, was not significantly related to recognition of fun (*β* = 0.651, *t* = 1.831, *95% CI* = − 0.051– 1.354).

#### Advisers’ recognition of fun

In the model for advisers’ recognition of fun, the fixed effects and random effect were identical to those in the model for advisers’ excitement recognition. A Wald Chi-Square test using the LMM revealed the significant main effects of turn (*χ*^2^(1) = 28.947, *p* < 0.001) and player’s heart rate (*χ*^2^(1) = 7.654, *p* = 0.006), with no other main effects reaching the threshold for statistical significance (all *p* > 0.05). The model showed that the intensity of the fun recognised by an adviser increased across turns (*β* = 0.105, *t* = 5.380, *95% CI* = 0.067–0.144) and with players’ higher heart rates (*β* = 0.050, *t* = 2.767, *95% CI* = 0.014–0.086). However, an adviser’s estimation of a player’s fun was not significantly related to that player’s subjective fun (*β* = − 0.033, *t* = − 0.569, *95% CI* = − 0.145–0.080), indicating that the adviser’s estimation of fun was incongruent with the player’s subjective fun. The adviser’s heart rate, which was associated with advisers’ excitement recognition, was not significantly related to recognition of fun (*β* = 0.017, *t* = 0.907, *95% CI* = − 0.020–0.054).

Unlike recognition of excitement, a player’s subjective fun and a bystander’s and adviser’s estimation of it, was generally incongruent. Moreover, there was no significant relationship between subjective or perceived fun and physiological synchrony.

## Discussion

Our findings show that first-person excitement experienced during cooperative Jenga was associated with physiological synchrony between players and advisers, and was not predicted solely by their own physiological responses. This indicates that the intensity of people’s display of their excitement is higher when their partner’s physiological response is synchronised with their own. Therefore, the relationship between emotional synchrony and intensity of subjective emotional experiences, which was reported on the basis of self-reported perceived synchrony^9^, is also observed when emotional synchrony is measured physiologically.

Our findings on bystanders’ emotion recognition indicate that perceived emotional intensity is higher when interacting people show congruent physiological emotional responses. Specifically, a bystander’s estimation of a player’s excitement increased with physiological synchrony between player and adviser. Interestingly, we found that this relation remained even when controlling for the effect of intensity and facial synchrony of smile expressions (i.e., intensity and interpersonal coherence of activities of the *zygomatic major*: AU12), a result obtained from an exploratory analysis that we added in accordance with reviewers’ comments. The LMM analysis revealed that bystanders’ greater perceived excitement was significantly associated with the physiological synchrony strength, even when the model included intensity and synchrony of the players’ and advisors’ smiles (in the model, the advisor’s facial activity and facial synchrony between player and advisor were marginally related to bystanders’ perceived excitement, see Supplementary Information for details). Therefore, the emotional intensity perceived by bystanders might be modulated not only by the congruency of emotional expressions^14,16^ but also by the synchronisation of the physiological dynamics of the interacting people. Although the mimicry of emotional expressions (e.g.,^7,27–29^) had little impact on the relationships between physiological synchrony and perceived emotion in our study, we did not investigate multiple perceptible signals possibly associated with physiological synchrony, such as synchronisation of motions or gestures^20,30–32^ or shared visual attention^33–35^. Further research is needed to identify perceptible signals associated with physiological synchrony and thereby shed light on how physiological synchrony has such a remarkable relationship with bystanders’ emotion recognition. For instance, simultaneous and continuous measurement of physiological synchrony, bodily synchronisation, and shared visual attention, when people jointly experience emotional events, will help us better understand the process by which certain perceptible social influences are associated with physiological synchronisation and also how they affect bystanders’ emotion recognition.

Unlike bystanders’ emotion recognition, that of the advisers was not associated with physiological synchrony. However, advisers’ perception of their partners’ excitement was related to their own heart rate. That is, people involved in an interaction tend to perceive their partner as more excited when their own physiological arousal is high. This finding seems to be consistent with the embodied cognition theory^36^ in which people tend to use their own physical state to estimate others’ emotional experiences (e.g.,^37,38^). It is possible that the cognitive processes involved in the interacting partner’s emotion recognition differ from those of third-party bystanders because the former is partly based on embodied cognition while the latter is partly based on a holistic perspective that captures interpersonal phenomena. To confirm this conjecture, however, simultaneously measuring advisers’ and bystanders’ physical states will be needed in future research.

Notably, heart rate synchrony was more evident in low-frequency bands associated with arterial pressure Mayer waves (IPC_LF_) than in high-frequency bands known as RSA (IPC_HF_), and the relationships between physiological synchrony and evaluation of emotional experiences were observed only in low- but not high-frequency bands. The magnitude of RSA is known to be related to respiratory changes; more specifically, it increases with increased tidal volume and decreased breathing frequency. However, heart rate variability at low frequencies is slower than respiration and is associated with arterial pressure (see^21^ for a review). Therefore, the physiological synchrony we observed, which was associated with the evaluation of emotional experiences, might reflect a synchrony of changes in arterial pressure rather than respiratory synchrony. Considering that RSA synchrony is associated with intensity of negative affect during marital conflict^22^, it may be possible that the synchrony in high- and low-frequency bands have different impacts depending on the type of emotion perceived under the different situations. Since our participants were limited to woman friends, further research is necessary to examine the relationship between perceived emotions and the heart rate synchrony in high- versus low-frequency bands in multiple contexts and relationships (e.g., cooperative/competitive situation and friends /couples).

Given that low-frequency heart rate oscillations, which occur in synchrony with arterial pressure Mayer waves, are thought to result from an oscillation in sympathetic vasomotor tone^39,40^, our findings suggest that the changes in sympathetic nervous system responses of interacting people tend to synchronise during cooperative joint tasks, and that the synchrony of sympathetic nervous system activities is associated with the recognition of excitement by both the self and third-party bystanders. It should also be noted that there is currently no evidence of a simple linear relationship between Mayer wave amplitude and mean sympathetic outflow^41^. Given this complexity of heart rate variability, future studies measuring multiple indices simultaneously such as respiratory rate, blood pressure, and heart rate in cooperative and confrontational situations are needed to understand the psychophysiological mechanisms underlying the association between physiological synchrony and perceived emotions.

Although, cooperative joint tasks in our study successfully induced players’ subjective fun (mean = 6.503, SD = 1.941) and subjective excitement (mean = 6.234, SD = 2.696), we could not find any associations between perceived fun and physiological synchrony. In addition, the estimated fun reported by bystanders and interacting partners was generally incongruent with players’ subjective fun. One of the reasons might be that the game situation that induced subjective excitement seemed to be somewhat common among individuals, while the situation that induced subjective fun varied greatly among individuals. For instance, some people reported experiencing fun when the tower was unstable, while others experienced fun when a block was smoothly removed. However, we are not yet able to draw any concrete conclusions on this point. Investigating differences among emotions in relation to physiological synchrony in future research would likely be fruitful for understanding its collective effects on emotions.

Taken together, our findings have shown that both the first-person subjective excitement and third-person recognition of excitement are associated with the heart-rate synchrony of interacting people in low-frequency oscillations, which reflects changes in sympathetic nervous system activities. Although further investigation will be necessary to clarify causal relationships between physiological synchrony and perceived emotion, given the James–Lange-type theories of emotion^42,43^, which suggest that physical responses are evoked prior to evaluations of feelings, one possible interpretation of the findings in the present study may be that physiological synchrony during joint tasks can lead to subjective experiences and recognition of greater excitement. Because both first- and third-person emotion recognition could have cumulative impacts on a group, it would be interesting to investigate how physiological synchrony contributes to changes in emotional mood in naturalistic groups, such as collective rituals or musical performances (e.g.^1,9,44,45^). The analysis of the temporal dynamics of both behavioural and physiological emotional responses developed through interpersonal interaction (e.g.^7^) will advance our understanding of the causal relationships that underlie psychophysiological links and group-level emotional phenomena such as crowd joy^46^ and collective emotional effervescence^10^.

## Methods

### Ethics statements

Study protocol was approved by the NTT Communication Science Laboratories Research Ethics Committee and was performed in accordance with the ethical standards set down in the 2013 Declaration of Helsinki. Written informed consent was obtained from all participants before beginning the experiment.

### Participants

To control the intimacy between the pairs who played Jenga and the observing bystanders, we recruited, on the same day, four women who were familiar with each other. Because we wanted to reduce the variations in heart rate caused by gender^47^, only women were recruited. Six units, each comprising two players and two bystanders, were created by changing combinations of the four people. The experiment was conducted for five days; thus, a total of 30 units, with combinations of 20 volunteers (aged 20–36, mean = 27.00 years, SD = 5.20), participated in the experiment. In each session, two players in a pair performed a cooperative joint task, and the remaining two participants in the same group were assigned as bystanders to evaluate the players’ emotions. There were a total of six daily sessions, taking a total of about 220 min. When the participants were recruited, they were informed that their heart rates and actions would be recorded by sensors and video cameras during the task. The participants who agreed with the procedure were asked to provide written informed consent. Participants received compensation (5000 JPY [45 USD]) for their participation.

### Cooperative joint task

The two participants in each pair were seated face-to-face while they played a cooperative block-stacking game (Jenga), alternating roles as player and adviser for each turn. The player removed one block in each turn from a tower constructed of 54 blocks. After removing the block, the player placed it on top of the tower, resulting in an increasingly unstable structure. Participant pairs were instructed to discuss and decide before playing (i.e., touching a block) which blocks to remove, to report their subjective experience after taking a turn as a player, and to estimate their partner’s subjective experience when they were acting an adviser. Players were allowed to poke a few blocks and estimate if they could remove them smoothly. Thus, in a typical case, after consulting with the adviser, the player would carefully poke one block, and then discuss with the adviser whether they could remove it. For analysis of physiological arousal, ECGs of the two participants were recorded during the task, and the action of removing a block was recorded by video camera (FDR-X3000R, Sony Inc.) at 30 frames per second from one side of the pair.

Furthermore, we set several rules for Jenga, because if the game is too easy, the need for cooperation is reduced. Each Jenga blocks was one of three colours: red, green, or yellow. The colour of the block that a player had to remove was determined by rolling a die (i.e., one-third probability). Each time a player rolled the die, they were allowed three trial touches to estimate whether the block could be removed. If the player tried three times and found it difficult to remove the third block, she had to roll the die again. Players were allowed to roll the die up to two times in each turn. Thus, players and advisers were required to make various judgments in a single turn. If, after rolling the second die, the player finally judged that no blocks could be removed, the turn was considered missed, and the role was switched to the next turn (i.e., “pass”). Passes occurred in less than 1% of the total turns (see Supplementary Table S1).

### Procedure

While all the participants were familiar with Jenga as a popular block game, they did not play frequently. Therefore, at the beginning of the experiment, to familiarise themselves with Jenga, four participants together practised pulling blocks from the tower several times. After being assigned roles, two paired participants were seated facing each other at a table with a Jenga tower on it, and two bystanders were seated each on one of the two remaining sides, facing each other (see Supplementary Fig. S1). The bystanders were seated at the same table as the player and adviser for the purpose of comparing second-person and third-person emotion recognition, and wore masks so that their facial expressions did not affect the emotional state of the player and the adviser. ECG transducer electrodes were attached to both participants in the pair. To synchronise ECG data with the recorded video, the experimenter clapped the clapperboard at the beginning of the joint task.

After each play, the player reported her subjective experience and the adviser and the bystanders reported their estimations of the player’s experiences using tablets. The tablets were placed so that the participants could not see the others’ ratings. The roles of player and adviser alternated in a pair throughout the 14 turns. If the tower fell before the 14th turn, the session ended.

### Subjective evaluation and estimation of emotional experience

After each play, the player was asked to report her subjective experience (i.e., “Did you feel excited? and “Did you have fun?”) and the adviser and the bystanders were asked to report their estimations of the player’s experience (“Did the player look excited?” and “Did the player look like she had fun?”). All feedback was recorded on tablets (Si03BF, SIAL Co., Ltd.) and was rated on an 11-point scale ranging from 0 (not at all) to 10 (extremely).

### Index of physiological responses during play

We recorded the ECGs using a BIOPAC system (MP150/ECG100C, BIOPAC Systems Inc.) to measure inter-beat intervals (IBIs). The sampling rate was 1000 Hz. R-wave detection was performed with AcqKnowledge 4.3 (BIOPAC Systems Inc.), then IBIs (in milliseconds) were calculated. The IBI data were pre-processed with MATLAB (MathWorks, Inc., Natick, MA) for subsequent analysis. The time series for IBIs were spline-resampled at 100 Hz, which was used for the analysis of physiological coupling. For analysis of physiological arousal, these interpolated IBIs were converted to heart rate values, which were expressed as beats per minute (BPM). Block-pull timing was detected from the recorded video; 25 s of interpolated IBIs and heart rates, 3 s before that timing, were used for analyses of physiological synchrony and arousal (see Fig. 1).

### Data treatment and analysis

ECG data from one pair were excluded because of equipment failure. There were problems with video recording for two pairs (e.g., dead battery). The data for a pair who collapsed the tower on the 4th turn was also excluded from the analysis because there were too few data points to analyse; a total of 26 pairs were included for analysis. There were two more cases in which the tower collapsed; because they occurred on the 12th and 13th turns, just before the final turn (i.e., 14th turn), we used data for up to one turn before the collapsed turns (i.e., 11th and 12th turns) for analysis (see Supplementary Table S1).

### Interpersonal physiological coherence (IPC)

IPC was estimated using WTC as the index of physiological synchrony between player and adviser. WTC is a method of measuring the correlation between two time-series data sets as a function of frequency and time^25,26^. We calculated the WTC of players’ and advisers’ IBIs using the wavelet toolbox in MATLAB (MathWorks, Inc., Natick, MA). The IPCs were calculated by averaged WTC values over time and high- (IPC_HF_: 0.15–0.40) or low-frequency bands (IPC_LF_: 0.04–0.15 Hz), excluding the cone of influence (COI) area.

To determine whether the IPCs were stronger because the social interaction happened between two participants who constituted an actual pair, we compared the IPCs of actual pairs with those of pseudo pairs. Pseudo pairs comprised two participants from different sessions, thus they were not paired during the task. Since participants in an actual pair alternated between the roles of player and adviser while performing the joint task, one participant in the pair played the odd-numbered turns and the other played the even-numbered turns. For comparison with actual pairs, pseudo pairs were all possible combinations of the participants playing in odd-numbered turns and the participants playing in even-numbered turns (see Supplementary Fig. S1).

## Supplementary Information

Supplementary Information.

## Data Availability

The datasets used in the current study are available from the corresponding author on reasonable request.
