# Supplementary material for: Interpersonal physiological synchrony is associated with first person and third person subjective assessments of excitement during cooperative joint tasks
Source: Sci Rep. 2021 Jun 15;11:12543. doi: 10.1038/s41598-021-91831-x (PMC8206359; doi:10.1038/s41598-021-91831-x)
Supplement: Supplementary file 1 — Supplementary Information. [file 41598_2021_91831_MOESM1_ESM.pdf]

Supplementary Information

Interpersonal physiological synchrony is associated with first person and third person subjective assessments of excitement during cooperative joint tasks

Aiko Murata[1*], Keishi Nomura[1,2], Junji Watanabe[1], and Shiro Kumano[1]

1. NTT Communication Science Laboratories, NTT Corporation, Atsugi, Japan

2. Graduate School of Education, The University of Tokyo, Tokyo, Japan

*Address correspondence to: aiko.murata.kb@hco.ntt.co.jp

Sample dynamics and sample size

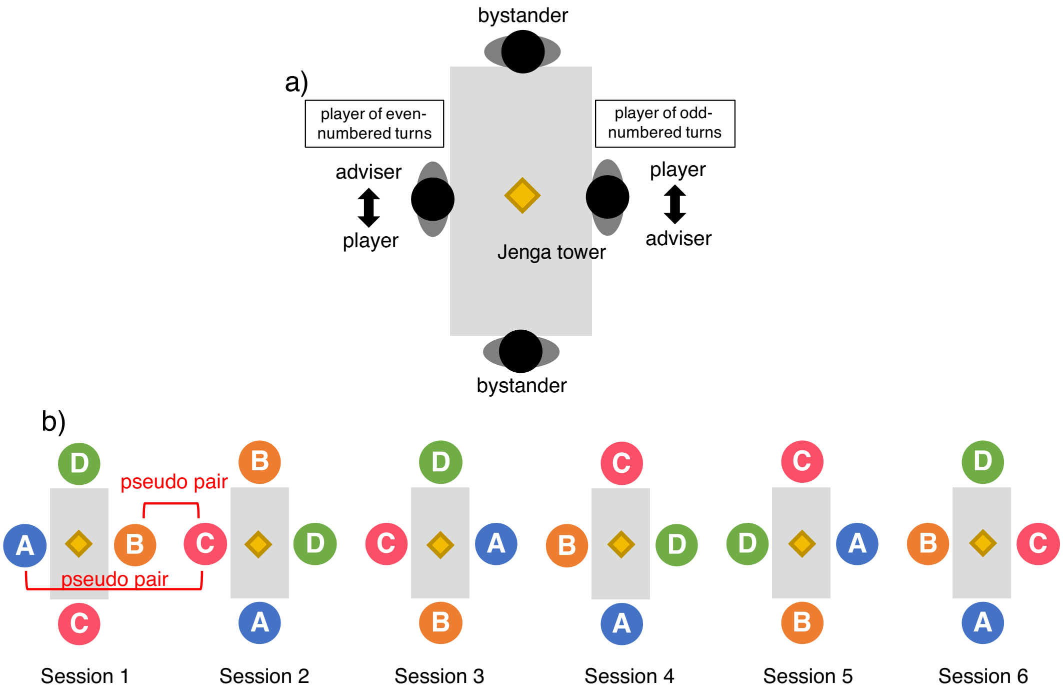

Figure S1. Sample dynamics of actual pairs and pseudo pairs

The seat configuration and pairings for one study day. A to D represent four participants attending six sessions. For each session, participants were assigned the roles: player of even-numbered turns, player of odd-numbered turns, or bystander. (a) Two participants playing Jenga were seated facing each other at a table, and two bystanders were seated at the other two sides of the table. (b) A unit with two players and two bystanders was created by changing the combinations of the four people. Pseudo pairs for IPC analysis were all possible combinations of the participants playing in odd-numbered turns and the participants playing in even-numbered turns. An example of a pseudo pair is shown in the figure.

Table S1. Number of turns per pair used in the analysis, and types of missing values. Turns that could not be used for analysis are indicated by an asterisk, and those that could be used are indicated by a check mark.

Turn:	1	2	3	4	5	6	7	8	9	10	11	12	13	14	Total number of turns
Pair 1	***1**	✓	✓	***1**	✓	✓	✓	✓	✓	✓	✓	✓	✓	✓	12
Pair 2	✓	✓	✓	✓	✓	✓	✓	✓	✓	✓	✓	✓	✓	✓	14
Pair 3	✓	✓	✓	✓	✓	✓	✓	✓	✓	✓	✓	✓	✓	✓	14
Pair 4	✓	✓	✓	✓	✓	✓	✓	***1**	***1**	✓	✓	✓	✓	✓	12
Pair 5	✓	✓	✓	✓	✓	✓	✓	✓	✓	✓	✓	✓	✓	✓	14
Pair 6	✓	✓	✓	✓	✓	✓	✓	✓	✓	✓	✓	✓	✓	✓	14
Pair 7	✓	✓	✓	✓	✓	✓	✓	✓	✓	✓	✓	✓	✓	✓	14
Pair 8	✓	✓	✓	✓	✓	✓	✓	✓	✓	✓	✓	✓	***2**	***2**	12
Pair 9	✓	✓	✓	✓	✓	✓	✓	✓	✓	✓	✓	✓	✓	✓	14
Pair 10	✓	✓	✓	✓	✓	✓	✓	✓	✓	✓	✓	✓	✓	✓	14
Pair 11	✓	✓	✓	✓	✓	✓	✓	✓	✓	✓	✓	✓	✓	✓	14
Pair 12	✓	✓	✓	✓	✓	✓	✓	✓	✓	✓	✓	✓	✓	✓	14
Pair 13	✓	✓	✓	✓	✓	✓	✓	✓	✓	✓	✓	✓	✓	✓	14
Pair 14	✓	✓	✓	✓	✓	✓	✓	✓	✓	✓	✓	✓	✓	✓	14
Pair 15	✓	✓	✓	✓	✓	✓	✓	✓	✓	✓	✓	✓	✓	✓	14
Pair 16	✓	✓	✓	✓	✓	✓	✓	✓	✓	✓	✓	✓	✓	✓	14
Pair 17	✓	✓	✓	✓	✓	✓	✓	✓	✓	✓	✓	***2**	***2**	***2**	11
Pair 18	✓	✓	✓	✓	✓	✓	✓	✓	✓	✓	✓	✓	✓	✓	14
Pair 19	✓	✓	✓	✓	✓	✓	✓	✓	✓	✓	✓	✓	✓	✓	14
Pair 20	✓	✓	✓	✓	✓	✓	✓	✓	✓	✓	✓	✓	***3**	***3**	12
Pair 21	✓	✓	✓	✓	✓	✓	✓	✓	✓	✓	✓	✓	✓	✓	14
Pair 22	✓	✓	✓	✓	✓	✓	✓	✓	✓	✓	✓	✓	✓	✓	14
Pair 23	***3**	✓	✓	✓	✓	✓	✓	✓	✓	✓	✓	✓	✓	✓	13
Pair 24	✓	✓	✓	✓	✓	✓	✓	✓	✓	✓	✓	✓	✓	✓	14
Pair 25	✓	✓	✓	✓	✓	✓	✓	✓	✓	✓	✓	✓	✓	✓	14
Pair 26	***3**	✓	✓	✓	✓	✓	✓	✓	✓	✓	✓	✓	✓	✓	13

Data unavailable for analysis because of the following reasons:
*1 The participant passed on their turn
*2 The tower collapsed
*3 ECG signal noise

The analysis of the effects of facial activities and synchrony of players and advisors on bystanders' perceived excitement

As we recorded the video during the task to specify the timing of pulling the block, the faces of the player and the adviser were also recorded. However, there were several facial activities that could not be captured because the face position and orientation dynamically changed during the task. Specifically, the activity of the upper faces sometimes could not be captured at the set camera angle. Despite the limited data on facial activities, and in accordance with the reviewers' comments, we examined whether the relationship between physiological synchrony and bystanders' perceived emotions (which we observed in our study) was over and above the effect of intensity and synchrony of facial activities.

Facial activities were analysed using OpenFace [1]. We used a 25-second period of interest from 28 to 3 seconds before removing a block, as well as IBIs. Interpersonal facial coherence (IFC) was estimated using WTC. It should be noted, however, that we needed to consider two problems: facial activity data were calculated as missing when the entire face went out of frame and reported as 0 when certain facial muscle activity was not captured at the set camera angle. Therefore, we set several limitations for facial data use. The data excluded from analysis were: 1) when both players' and advisors' facial activity data were not available for more than 5 consecutive seconds, or 2) the value of 0 was continuous for more than 2.5 seconds (in other words, when at least 50% of the data could not be used for analysis).

Since cooperative joint tasks in our study successfully induced subjective fun (mean = 6.503, SD = 1.941) and subjective excitement (mean = 6.234, SD = 2.696), we analysed facial activities related to joy and surprise, specifically, AU12 (*Zygomatic Major*) for the joy expression, and AU1 (*Frontalis, pars medialis*), AU2 (*Frontalis, pars lateralis*), AU5

(*Levator palpebrae superioris*) for the surprised expression. Figure S2 shows histograms of the mean activity and IFC of each muscle. As shown, both the activity and synchrony of AU12 were remarkably higher than those of the other AUs. Therefore, we used AU12 to evaluate the effect of facial synchrony and activity on the perceived emotion. In the LMM for bystanders' excitement recognition, in addition to IPC, means and standard deviations of players' and advisers' heart rates, play duration, turn, session, and players' subjective emotion, facial synchrony, and facial activity (AU12 mean activity was calculated for each trial) were entered as fixed effects. A Wald Chi-Square test using the LMM revealed the significant main effects of the player's subjective excitement ($\chi 2(1) = 12.568$, $p < 0.001$), turn ($\chi 2(1) = 46.743$, $p < 0.001$), the player's mean heart rate ($\chi 2(1) = 5.257$, $p = 0.022$), and physiological synchrony in the low-frequency band (IPC_{LF}: $\chi 2(1) = 6.956$, $p = 0.008$). Play duration ($\chi 2(1) = 3.579$, $p = 0.059$), facial synchrony (IFC_{AU12}: $\chi 2(1) = 3.066$, $p = 0.080$) and adviser's facial activity (mean activity of AU12: $\chi 2(1) = 3.827$, $p = 0.050$) were marginally significant. No other main effects were significant. Although the effect was moderate, greater facial synchrony was positively related to the bystanders' perceived emotion intensity ($\beta = 3.141$, $t = 1.751$, *95% CI* = -0.388−6.671), in line with the previous study [2]. Notably, physiological synchrony was significantly associated with perceived excitement ($\beta = 1.830$, $t = 2.638$, *95% CI* = 0.453−3.200), even when the model included the effects of facial activity intensity and synchrony.

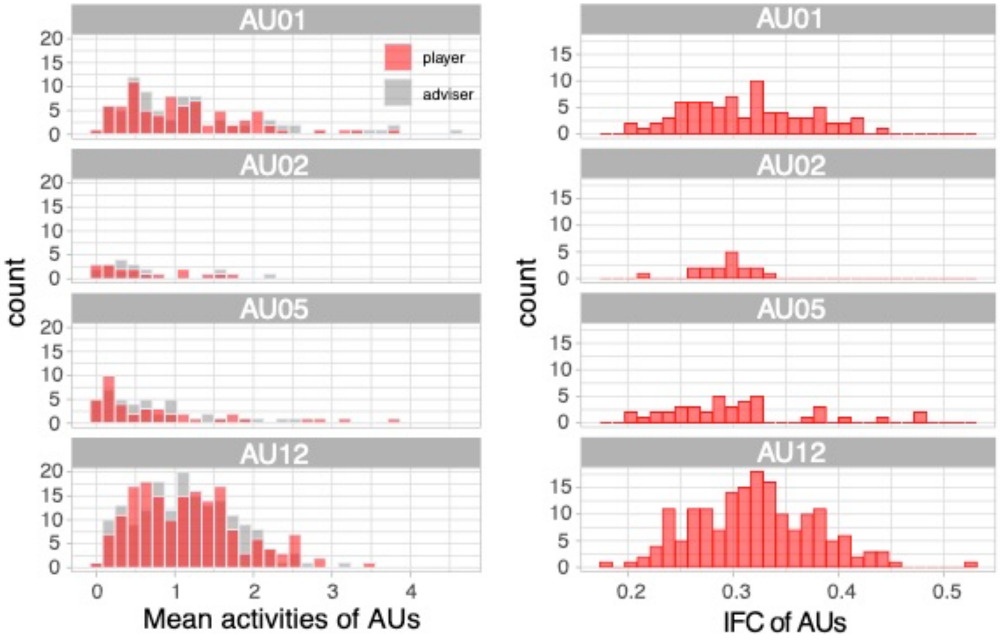

Figure S2. Histograms of the mean activity and IFC of each AU (muscle).

Phase angle analysis of physiological synchrony

To assess the leader-follower relationship of physiological synchrony between players and advisers, we calculated the relative phase from players to advisers at each time point (a 25-second period of interest; 2500 time points in total) in the wavelet coherence, which indicated a lag in physiological synchrony. Since phase can only be meaningfully interpreted when there is sufficiently high coherence, we decided to use phase data when coherence exceeded 0.5. Figure S3 shows a phase-frequency histogram. If the player's heart rate increased/decreased before the adviser's heart rate increased/decreased (i.e., the adviser's heart rate followed the player's heart rate), the peak of the phase showed a positive value. As shown in Supplementary Figure S3, there was no clear leader-follower relationship between players and advisers in our study, because the peak of the phase was around 0°.

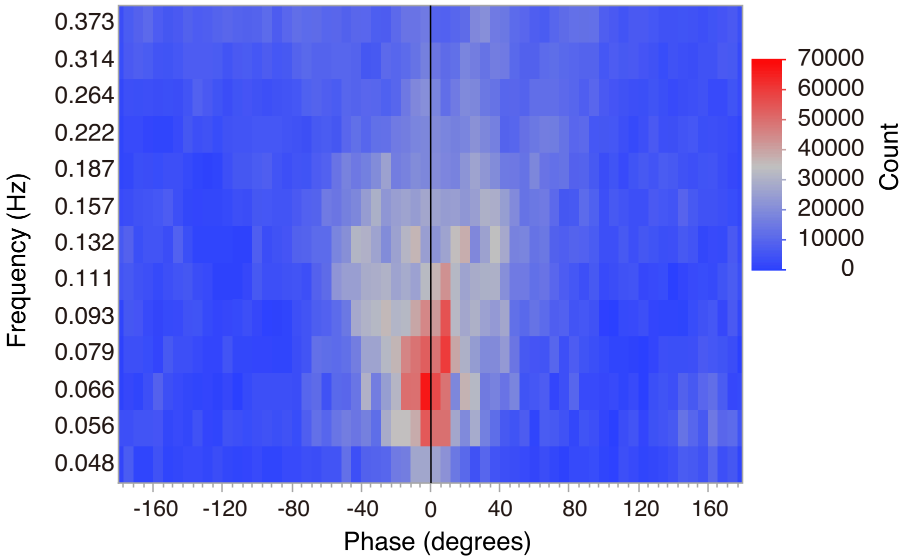

Figure S3. A phase-frequency histogram of physiological synchrony

References

1. Baltrušaitis, T., Robinson, P., & Morency, L. P. Openface: an open source facial behavior analysis toolkit. *In 2016 IEEE Winter Conf Appl Comput Vis. (WACV)*. 1-10 (2016, March).
2. Hess, U., Blaison, C., & Kafetsios, K. Judging facial emotion expressions in context: The influence of culture and self-construal orientation. *J Nonverbal Behav.* **40**(1), 55-64 (2016).
